# High-Intensity Strength Training Improves Function of Chronically Painful Muscles: Case-Control and RCT Studies

**DOI:** 10.1155/2014/187324

**Published:** 2014-02-23

**Authors:** Lars L. Andersen, Christoffer H. Andersen, Jørgen H. Skotte, Charlotte Suetta, Karen Søgaard, Bengt Saltin, Gisela Sjøgaard

**Affiliations:** ^1^National Research Centre for the Working Environment, Lersø Parkalle 105, 2100 Copenhagen, Denmark; ^2^Section of Clinical Physiology and Nuclear Medicine, Department of Diagnostics, Glostrup Hospital, University of Copenhagen, 2600 Glostrup, Denmark; ^3^Institute of Sport Science and Clinical Biomechanics, University of Southern Denmark, Campusvej 55, 5230 Odense M, Denmark; ^4^Copenhagen Muscle Research Centre, University of Copenhagen, 2100 Copenhagen, Denmark

## Abstract

*Aim*. This study investigates consequences of chronic neck pain on muscle function and the rehabilitating effects of contrasting interventions. *Methods*. Women with trapezius myalgia (MYA, *n* = 42) and healthy controls (CON, *n* = 20) participated in a case-control study. Subsequently MYA were randomized to 10 weeks of specific strength training (SST, *n* = 18), general fitness training (GFT, *n* = 16), or a reference group without physical training (REF, *n* = 8). Participants performed tests of 100 consecutive cycles of 2 s isometric maximal voluntary contractions (MVC) of shoulder elevation followed by 2 s relaxation at baseline and 10-week follow-up. *Results*. In the case-control study, peak force, rate of force development, and rate of force relaxation as well as EMG amplitude were lower in MYA than CON throughout all 100 MVC. Muscle fiber capillarization was not significantly different between MYA and CON. In the intervention study, SST improved all force parameters significantly more than the two other groups, to levels comparable to that of CON. This was seen along with muscle fiber hypertrophy and increased capillarization. *Conclusion*. Women with trapezius myalgia have lower strength capacity during repetitive MVC of the trapezius muscle than healthy controls. High-intensity strength training effectively improves strength capacity during repetitive MVC of the painful trapezius muscle.

## 1. Introduction

Neck/shoulder pain is a frequent condition in the working population [1] and pain, tightness, and tenderness of the upper trapezius muscle—trapezius myalgia—are the most common type of chronic neck/shoulder pain [2, 3]. In a study among elderly female computer workers with neck/shoulder pain 38% were clinically diagnosed with trapezius myalgia [3]. In another study among office workers with frequent neck/shoulder pain three out of four experienced tenderness by palpation of the upper trapezius muscle [4]. Trapezius myalgia is associated with pathophysiological changes, such as increased occurrence of ragged red fibers, moth eaten fibers, and cytochrome oxidase negative type I fibers [5, 6]. Using the microdialysis technique, increased concentration of the algesic substance serotonin during work, stress, and rest has also been shown [7]. Recent studies combining muscle biopsies and microdialysis with gel electrophoresis and mass spectrometry identified several proteins involved in inflammatory processes in myalgic trapezius muscles compared with healthy controls [8, 9]. Women with trapezius myalgia also have reduced capillarization of type I muscle fibers [10], increased proportion of poorly capillarized type I megafibers [11], impaired regulation of microcirculation locally in the painful trapezius muscle [12–14], and reduced capacity of carbohydrate oxidation [15]. These factors lead to elevated anaerobic metabolism and fatigue during repetitive work [16, 17].

In addition, trapezius myalgia negatively impacts muscle functioning. Compared with healthy controls, women with trapezius myalgia show decreased maximal voluntary force, rapid force, motor control, endurance, and neck flexibility [18–22]. Trapezius myalgia has also been associated with an inability to properly relax the muscle between repeated contractions [23], which may aggravate development of fatigue. Thus, prevention as well as rehabilitation of neck/shoulder pain has focused on improving different components of physical function such as the ability to relax the muscles using biofeedback training [24, 25], muscle coordination training [26], muscle strength using high-intensity resistance training [26–28], endurance using repeated low-intensity contractions [26, 27], fitness training [29], and flexibility using stretching [27]. Among these training modalities strength training appears particularly effective in reducing pain and increasing muscle strength in trapezius myalgia [28]. However, transfer effects of strength training to improve work capacity of the painful trapezius muscle have only been scarcely investigated [26]. This is a potential important aspect, as daily job tasks require repetitive muscle contractions inducing a certain level of fatigue, for example, repetitive arm movements during assembly line work or keyboard typing. Studying the effect of strength training on strength-endurance during repetitive and fatiguing contractions therefore seems pertinent.

A contrasting approach to specific strength training could be to train large muscles distant from the painful site, for example, by general fitness training performed as leg cycling. Previous research in other populations has indicated physiological adaptations in sites distant from the trained muscles [30, 31]. Increased blood flow to the forearms [32] and nonworking limb [33] has also been shown in response to leg exercise. Based on these studies, general fitness training performed as leg cycling may also improve endurance of the painful trapezius muscle.

The aims of the present study were (1) in a case-control design to compare muscle function during fatiguing muscle contractions in women with trapezius myalgia and healthy controls and (2) in a randomized controlled trial to investigate the effect of contrasting types of physical rehabilitation in women with trapezius myalgia on muscle function during fatiguing muscle contractions. Additionally, analyses of electromyographic recordings and muscle biopsies were included to investigate neural and muscular adaptations. We hypothesized that specific strength training and general fitness training would be superior to the reference intervention with regard to improved muscle function.

## 2. Methods

### 2.1. Study Design and Participants

The study design, recruitment of participants, randomization of 48 women, and main results have been described in detail previously [14, 28]. In the present case-control study, 42 women with trapezius myalgia (MYA, mean ± SD: 44 ± 8 yrs, 165 ± 6 cm, 72 ± 15 kg, and days with neck pain during previous year 219 ± 19 days) and 20 women comparable with regard to job-type, age, weight, and height but without neck muscle complications (CON, mean ± SD: 45 ± 9 yrs, 167 ± 6 cm, 70 ± 11 kg, and days with neck pain during previous year 5 ± 6 days) participated. Exclusion criteria were serious conditions such as previous trauma, life threatening diseases, whiplash injury, cardiovascular diseases, or arthritis in the neck and shoulder. All participants were recruited from workplaces with monotonous and repetitive work tasks, mostly office- and computer-work. All females in MYA were clinically diagnosed with trapezius myalgia, where the main criteria for a positive diagnosis were (1) chronic pain in the neck area, (2) tightness of the upper trapezius muscle, and (3) palpable tenderness of the upper trapezius muscle [28]. In the 10-week intervention study, the 48 women with trapezius myalgia were randomly allocated in balanced design accounting for similar age, BMI, and neck/shoulder trouble to three different 10-week interventions, but 6 dropped out initially in the REF group resulting in 42 women in this study. The random allocation was concealed; that is, the researcher who determined the eligibility of the subjects was unaware which group a subject was to be allocated to. Unfortunately, the timewise successive balanced recruitment resulted in a somewhat smaller REF group, for example, due to withdrawal of 6 participants who initially stated they would volunteer for the study.

All subjects were informed about the purpose and content of the project and gave written informed consent to participate in the study, which conformed to the Declaration of Helsinki and was approved by the Local Ethical Committee (KF 01-138/04). The study was registered in the International Standard Randomised Controlled Trial Number Register (ISRCTN87055459).

### 2.2. Measurements and Data Acquisition

**Figure 1:**
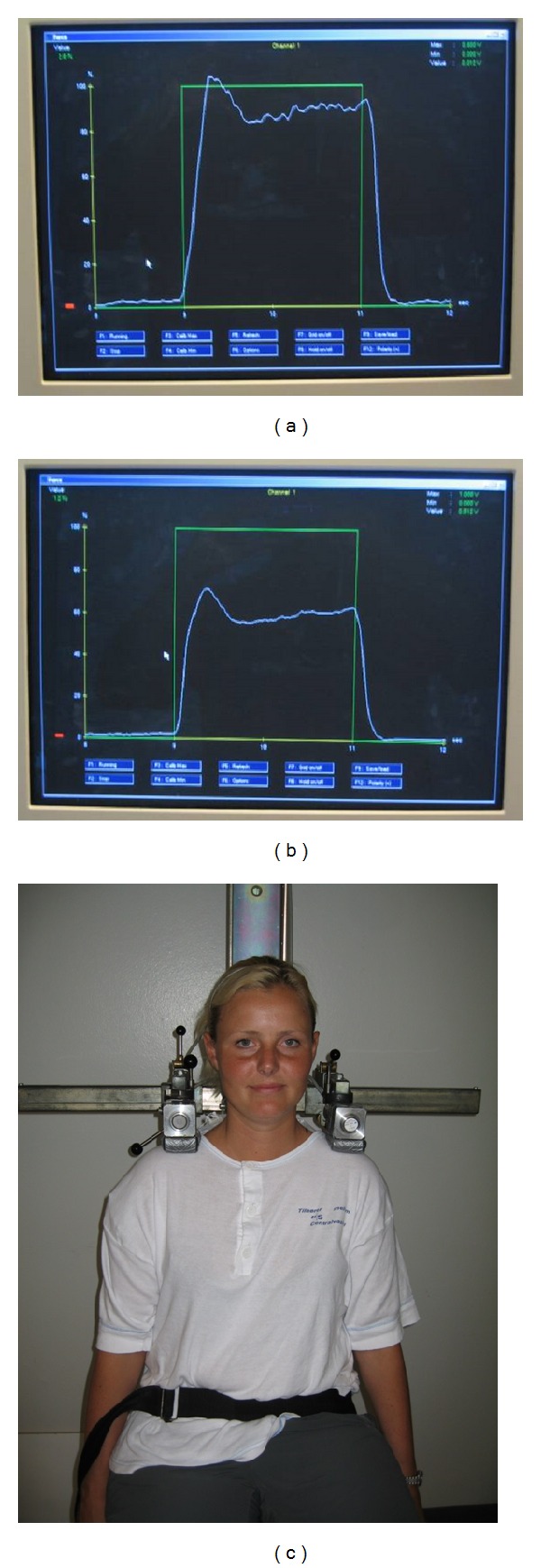
Illustration of experimental setup, the square wave signal (green tracing), and a typical force signal (white tracing) in the beginning (left) and during the end (right) of the 100 consecutive contractions.

*Muscle endurance* was measured during consecutive isometric shoulder elevations using a custom-built setup with a steel frame, a chair, and attached force dynamometers (Figure 1). The participant was sitting upright in the height-adjustable chair, and two Bofors dynamometers were placed bilaterally 1 cm medial to the lateral edge of the acromion with the direction of force being upward [34]. Prior to the endurance test, the participant performed 3 maximal voluntary isometric contractions (MVC) to determine peak force. The participant was then instructed to perform 100 consecutive cycles of 2-second MVC followed by 2-second relaxation. Figure 1 shows the square wave on the computer screen providing visual feedback instructing the participant when to contract and when to relax, with the target value of the square wave set to the peak force determined prior to the endurance test. The participant was instructed to contract as hard and fast as possible when the signal of the square wave went from zero to max and to relax immediately and completely when the signal went from max to zero. The 100 contractions were completed in 6 minutes and 40 seconds.

*Electromyography* (EMG) signals were recorded synchronously from the upper trapezius muscle with a bipolar surface EMG configuration (Neuroline 720 01-K, Medicotest A/S, Ølstykke, Denmark) and an interelectrode distance of 2 cm [35]. The electrodes were positioned according to SENIAM guidelines [36]. The skin was abraded prior to applying the electrodes to ensure an impedance of less than 10 kΩ (typically the impedance was 1-2 kΩ). If the impedance was higher than 10 kΩ the procedure was repeated until impedance was less than 10 kΩ. The EMG electrodes were connected directly to small preamplifiers located near the recording site. The raw analogue EMG signals were led through shielded wires to instrumental differentiation amplifiers, with a bandwidth of 10–400 Hz and a common mode rejection ratio better than 100 dB. Force and EMG signals were sampled synchronously at 1000 Hz using a 16-bit A/D converter (DAQ Card-Al-16XE-50, National Instruments, USA) and stored on a laptop for further analysis.

### 2.3. Data Analysis

Data were analyzed only for the signals from the most painful shoulder. In case of similar pain intensities in both shoulders the dominant side was used for the analyses. During later offline analyses, the force signal was low-pass filtered at 10 Hz, and then three parameters were calculated for each of the 100 MVCs: (1) peak force, determined as the maximal force value within each cycle, (2) rate of force development (RFD) determined as the maximal positive slope over 100 msec of the force signal during the beginning of each MVC, and (3) rate of force relaxation (RFR) determined as the maximal negative slope over 100 msec of the force signal during the end of each MVC. All RFR values were subsequently multiplied by −1 to ease comparison with RFD.

Likewise, the raw EMG signals were filtered using linear EMG envelopes, which consisted of (1) high-pass filtering at 10 Hz, (2) full-wave rectification, and (3) low-pass filtering at 10 Hz. The filtering algorithms were based on a fourth-order zero phase lag Butterworth filter [37]. From the filtered EMG signal the following parameter was calculated for each of the 100 MVCs: (1) peak EMG, determined as the maximal value of the filtered signal during the top phase of the square signal, that is, when muscle force is peaking, (2) integrated EMG during the first 1/2 second of the top phase of the square signal, that is, during the very beginning of muscle contraction, and (3) resting EMG determined as the average value of the filtered signal during the mid 1/2 second of the bottom phase of the square signal, that is, during relaxation between contractions.

The power spectral density of the EMG signals was calculated as the median power frequency (MPF) in epochs of 1000 ms in the midphase of each of the 100 contractions. The power density spectra were estimated by Welch's averaged, modified periodogram method in which each epoch was divided into eight Hamming windowed sections with 50% overlap.

### 2.4. Muscle Biopsies

Using the needle biopsy technique, muscle biopsies were obtained ultrasound guided from the upper trapezius muscle at the midbelly between the 7th cervical vertebrae and the acromion. The tissue samples were mounted with Tissue-Tek within 2-3 min, frozen in isopentane precooled with liquid nitrogen, and stored in a freezer at –80°C until processed. All biopsy samples were given a unique identification number and blinded. Transverse serial sections (10 *μ*m) of the embedded muscle biopsy were cut in a cryostat (Microm, Germany) (22°C) and mounted on glass slides. Standard ATPase analysis was performed after preincubation at pH values of 4.37, 4.61, and 10.30 [38]. The biopsy sections were visualized on a computer screen using a Carl Zeiss light microscope (Zeiss Axiolab), a JVC high-resolution color digital camera (JVC, TK-C1381EG), and an 8-bit Matrox Meteor Framegrabber (Matrox Electronic Systems, Quebec, Canada). Quantitative analysis of all muscle samples for fiber type percentage, fiber cross-sectional area (CSA), capillaries per fiber (CAF), and capillaries per fiber CSA (CAFA) was performed using a digital image analysis program (TEMA 1.04, Scanbeam, Hadsund, Denmark). All values are reported for types I and II fibers separately. The results on fiber type percentage (case-control study), fiber type area percentage (intervention study), CSA (case-control and intervention study), and CAF and CAFA (case-control study) have been reported previously [11, 39] and are reported here only for comparison with the results from the endurance test. Results from the intervention study for fiber type percentage, CAF, and CAFA have not been reported previously and are reported here as original data.

### 2.5. Interventions

After the case-control study, the 42 women with trapezius myalgia were randomly allocated to three different 10-week interventions. The specific strength training group (SST, *n* = 18) performed five dumbbell exercises specifically for the shoulder and neck muscles (shoulder abduction, shoulder elevation, 1-arm row, reverse flyes, and upright row) for 20 min three times a week. The high level of activity of the neck and shoulders muscles using these exercises has been documented elsewhere [40]. Three of the five exercises were performed during each session for three sets of each exercise using relative loadings of 8–12 repetitions maximum (RM). The strength training schedule followed principles of progressive overload and periodization as recommended by the ACSM [41]. The general fitness training group (GFT, *n* = 16) performed leg-bicycling in an upright position with relaxed shoulders on a stationary ergometer at relative loadings of 50% to 70% of maximal oxygen uptake for 20 min three times a week. The loading was estimated based on relative workload = (working heart rate − resting heart rate)/(max heart rate − resting heart rate), where resting heart rate was set to 70 bpm and max heart rate was estimated as 220 − age [42]. The reference group (REF, *n* = 8) received information concerning health promotion for one hour per week but were not offered any physical training. Unfortunately, the timewise successive balanced recruitment resulted in a somewhat smaller REF group compared with the two other groups, for example, due to withdrawal of participants who initially stated they would volunteer for the study.

### 2.6. Statistics

In the case-control study, differences between MYA and CON were tested with two-way ANOVA (Proc Mixed of SAS). The dependent variables were peak force, RFD, RFR, and peak EMG, respectively. Independent fixed factors included in the model were *group* (MYA, CON), *number* (100 contractions), and *group by number* interaction.

**Figure 2:**
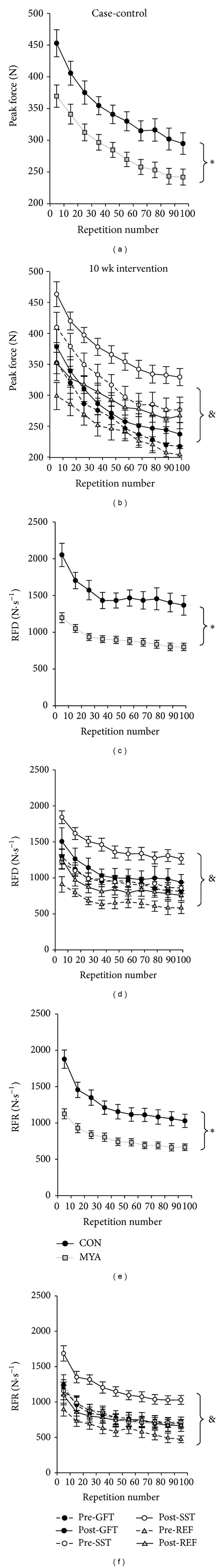
Case-control (left) and intervention (right) results of peak force (top), rate of force development (RFD) (mid), and rate of force relaxation (RFR) (bottom). *Significant *group* effect; *P* < 0.001 and *P* < 0.01.

In the intervention study, differences over time between the three groups were tested with repeated measures two-way ANCOVA (Proc Mixed of SAS). The dependent variables were the changes from baseline to follow-up in peak force, RFD, RFR, and peak EMG, respectively. Independent fixed factors included in the model were *group* (GFT, SST, and REF), *number* (100 contractions), and *group by number *interaction. The baseline value of the dependent variable was included as a covariate due to the numerical differences visualized in Figure 2. Participant was entered in the model as a nested random effect using the *repeated* statement.

The alpha level was set to 0.05, and results are reported as means ± SE in the figures and as means and 95% confidence intervals in the text.

Finally, when statistically significant changes were found we calculated effect sizes as Cohen's *d* (difference from baseline to follow-up divided by the pooled standard deviation at baseline) [43]. According to Cohen, effect sizes of 0.20 are small, 0.50 moderate and 0.80 large.

## 3. Results

### 3.1. Case-Control Study

**Figure 3:**
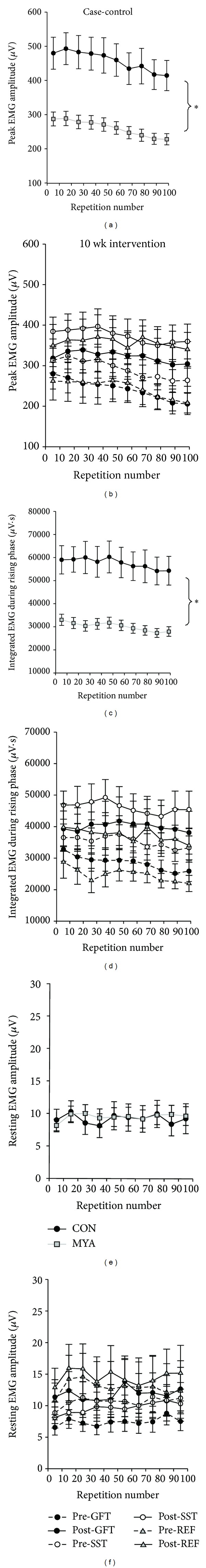
Case-control (left) and intervention (right) results of peak EMG (top), integrated EMG during the rising phase of contraction (mid), and resting EMG (bottom). *Significant *group* effect; *P* < 0.001 and *P* < 0.01.

**Figure 4:**
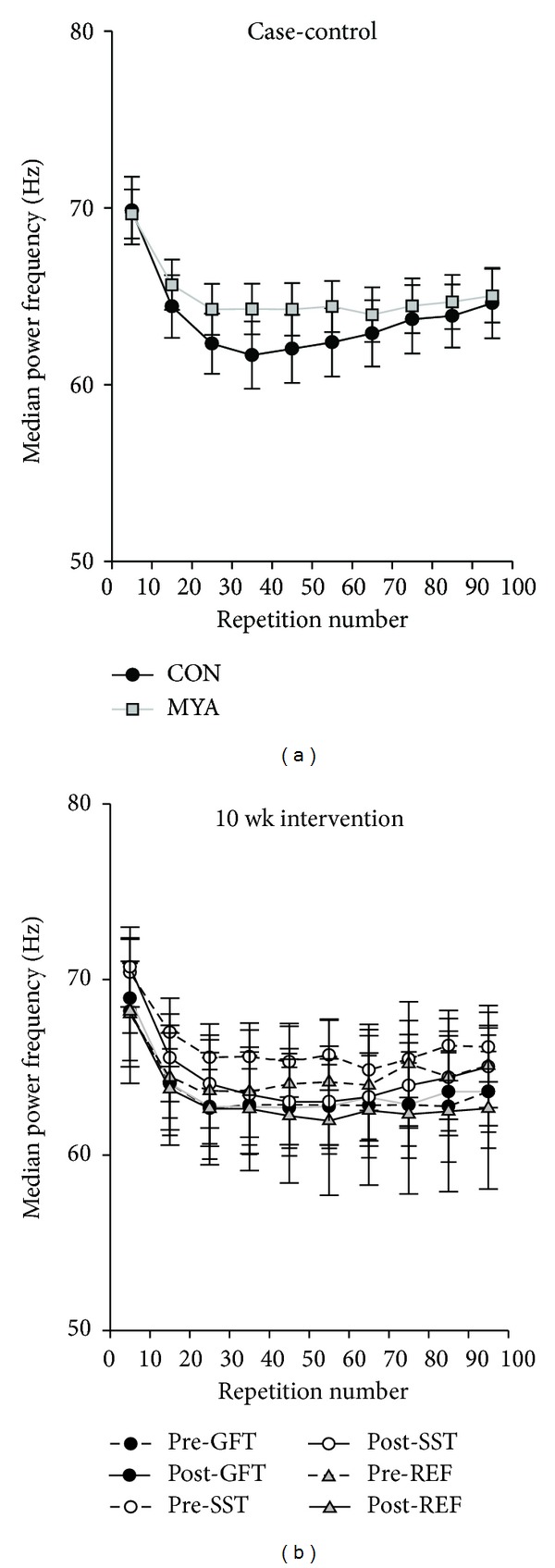
Case-control (left) and intervention (right) results of median power frequency (MPF) of the EMG signal.

There was a significant *group* effect for all three force parameters (*P* < 0.001). Post hoc analyses showed that peak force, rate of force development, and rate of force relaxation were lower in MYA compared with CON (Figure 2, *left*). There was a significant *group* effect for two of the four EMG parameters. Post hoc analyses showed that peak EMG and rate of EMG rise were lower in MYA compared with CON (Figure 3, *left*). There was no significant *group* effect for resting EMG (Figure 3, *left*) or MPF (Figure 4, *left*). There was no *group by number* interaction; that is, the slope over the 100 repetitions did not significantly differ between the two groups, although there was a borderline *group by number* interaction for MPF (*P* = 0.05).

Capillarization per fiber as well as per fiber area did not significantly differ between MYA and CON, neither for type I nor type II fibers (Table 1).

### 3.2. Intervention Study

There was a significant *group* effect for the change from baseline to follow-up for all three force parameters (*P* < 0.01–0.001). Post hoc analyses showed that the strength training group improved significantly more than the two other groups for peak force (SST versus GFT 70 N [95% CI 35–105], SST versus REF 50 N [95% CI 7–93]), rate of force development (SST versus GFT 346 N·s^−1^ [95% CI 97–595], SST versus REF 464 N·s^−1^ [95% CI 156–772]), and rate of force relaxation (SST versus GFT −378 N·s^−1^ [95% CI −233–(−)523], SST versus REF −323 N·s^−1^ [95% CI −143–(−)503]) (Figure 2, *right*). The effect sizes for these changes in the SST group were 0.61, 0.96, and 0.90 for peak force, rate of force development, and rate of force relaxation, respectively. Thus, the effects of SST can be considered moderate (peak force) to large (rate of force development and rate of force relaxation).

There was no significant *group* effect for the change from baseline to follow-up for any of the EMG parameters (Figure 3, *right,* and Figure 4, *right*). There was no *group by number* interaction for the change from baseline to follow-up; that is, the change in the slope over the 100 repetitions did not significantly differ between the three intervention groups.

Capillarization per fiber as well as muscle fiber cross-sectional area increased significantly in the SST group for type II fibers (*P* < 0.05) and tended to increase for type I fibers (*P* < 0.10). In SST, capillarization per fiber area remained unchanged in both types I and II fibers (Table 1). No significant changes occurred in the two other groups.

### 3.3. Test-Retest Reliability

Test-retest reliability of the outcome measures was not determined prior to the study. However, we performed reliability analyses after the study using the data from the two groups that did not respond to the intervention, that is, the GFT and REF groups. For these analyses the values for each subject were averaged for the 100 contractions at baseline and follow-up, respectively. The intraclass correlation coefficient (ICC) between baseline and 10-week follow-up was 0.85 for peak force, 0.70 for rate of force development, and 0.85 for rate of force relaxation.

### 3.4. Post Hoc Power Analysis

Due to the nonsignificant changes in the EMG parameters we performed a post hoc power analysis using the EMG data (averaged for the 100 contractions for each subject) from the two groups that did not respond to the intervention, that is, the GFT and REF groups. At baseline peak EMG was 244 uV (mean value) and the standard deviation of the change from baseline to follow-up was 139 uV. Requesting 80% power to detect 20% difference from baseline to follow-up with a type I error probability of 5% would then require 66 subjects in each group. Correspondingly, with only 14 subjects in each group the power to detect 20% difference is only 20.8%; that is, it is likely that 4 in 5 studies with similar sample size would report null findings.

## 4. Discussion

The main findings of the present study were (1) that women with trapezius myalgia showed reduced force capacity during repetitive maximal contractions of the trapezius muscle compared with healthy controls (case-control study) and (2) that 10 weeks of high-intensity specific strength training led to improved force capacity of the trapezius muscle in women with trapezius myalgia. By contrast, leg cycling did not improve trapezius muscle function. These results along with neural and muscular findings are discussed in the following.

In the case-control study, peak force, rate of force development, and rate of force relaxation as well as peak EMG and integrated EMG during the rising phase of contraction were lower in women with trapezius myalgia compared with healthy controls. We have previously shown—in the same group of subjects—that peak force and rate of force development during a single maximal voluntary contraction are lower in women with trapezius myalgia [21]. In the present study we elaborate on these findings by showing that all these force parameters are lower throughout all 100 contractions. However, the slopes of curves for the peak force values did not differ between the groups. This finding suggests that lower strength-endurance capacity in women with trapezius myalgia is related to the generally lower level of muscle strength—generally requesting a higher relative load during daily life and occupational tasks [14]—rather than faster development of fatigue.

Some of our case-control results contrast previous findings. In contrast to the findings by Larsson and coworkers [10] we did not find reduced capillarization of type I muscle fibers in women with trapezius myalgia compared with healthy controls. This is surprising since we have also in previous studies found a decreased oxygenation in women with trapezius myalgia compared to healthy controls during a more functional and submaximal test using a pegboard task [14]. This may suggest that impaired regulation of microcirculation, rather than reduced capillarization, may contribute to development of pain and fatigue during repetitive work tasks in women with trapezius myalgia.

Also, based on the EMG measurements we found that women with trapezius myalgia and healthy controls had similar ability to relax the muscles, that is, the same level of EMG amplitude, between repeated contractions, which contrast the EMG findings by Elert and coworkers [23]. However, there are methodological differences between the studies which make direct comparison difficult. Elert and coworkers used dynamic contractions and defined the ability to relax the trapezius as the ratio between EMG amplitude of the eccentric (relaxation phase) and concentric phase (contraction phase) of the 100 contractions. As a ratio depends both on the numerator and denominator it can increase simply by decreased maximal EMG during each concentric contraction, which makes the results by Elert and coworkers difficult to interpret.

Different types of normalization of the EMG signal are typically performed to avoid the inherent variance associated with EMG amplitude. By contrast, we did not normalize the EMG amplitude to a maximal voluntary contraction (MVC) as neck/shoulder pain inhibits central drive and thereby the EMG signal during maximal contraction [21, 22, 44]. Thus, normalizing the EMG signal to a maximal contraction would give erroneous normalized values when comparing individuals with and without pain and also when comparing data before and after an intervention that decreases pain. On the other hand, comparing nonnormalized EMG amplitudes between groups is also associated with limitations, such as variance due to electrode placement and thickness of subcutaneous fat. Therefore we carefully placed the EMG electrodes at the same part of the muscle between participants and test sessions by using the seventh cervical vertebrae and the acromion as reference points in accordance with SENIAM guidelines [36]. Further, we have previously reported that there was no difference between MYA and CON in thickness of the subcutaneous layer of fat above the trapezius muscle, and neither did this change significantly during the 10-week intervention period [45]. Thus, our results indicate that women with trapezius myalgia have the same ability to relax between muscle contractions.

In spite of similar resting EMG between contractions, the force measurements showed that relaxation from maximal to zero muscle contraction was slower, that is, lower rate of force relaxation, in women with trapezius myalgia than healthy controls. Thus, although women with trapezius myalgia have the ability to fully relax the muscles, it takes longer than in healthy controls. Although speculatively, this may be related to altered Ca^2+^ kinetics of myalgic muscles. Interestingly, Green and coworkers found a compromised sarcoplasmic reticulum Ca^2+^-ATPase activity, Ca^2+^-uptake, and Ca^2+^-release in a case-series study involving women with myalgia [46]. A slower relaxation of myalgic muscles may have negative implications for high pace work tasks where the time for recovery between contractions is minimal.

The intervention study showed that high-intensity strength training improves both maximal and rapid force capacity as well as the ability to rapidly relax—that is, rate of force relaxation—after each contraction. We have previously reported a marked reduction (79%) of pain along with increased maximal and rapid force capacity of single maximal voluntary contractions in response to the 10-week high-intensity strength training intervention [28, 39, 47]. The present study elaborates on these findings by showing that muscle function is improved during repetitive maximal muscle contractions. This may have important practical implications as many job tasks require repetitive muscle contractions, such as keyboard typing or repetitive arm movements during assembly line work. Further, previous analyses have shown increased carbohydrate oxidative capacity after specific strength training in women with trapezius myalgia [15], which may also help to explain the ability to sustain a high force development during repetitive contractions. Apart from improved muscle function during repetitive job tasks, the present study also demonstrated significant myofiber hypertrophy and increased capillarization in response to 10 weeks of specific strength training. Thus, these muscular adaptations may partly explain the present findings, although we cannot exclude the existence of neural adaptations mechanisms. Despite the fact that neither of the EMG parameters changed significantly between the intervention groups, the strength training group had the numerically highest EMG amplitude values at 10-week follow-up. Post hoc power analysis showed that 66 subjects in each group would be required to detect a 20% difference in peak EMG between groups from baseline to follow-up. Therefore, the present study was underpowered to detect significant changes due to the inherent high variability of the EMG measurements, making significant results more difficult to obtain with a small sample size.

Median power frequency of the EMG signal decreased as expected during fatiguing contractions (Figure 4). This was evident both in healthy controls and women with trapezius myalgia at baseline and follow-up. This validates that the endurance test induces muscle fatigue both among healthy individuals and individuals with pain.

We also included a general fitness training group who performed leg cycling while relaxing the shoulders. Previous research in other populations has shown physiological adaptations in sites distant from the trained muscles [30, 31]. Increased blood flow to the forearms [32] and nonworking limb [33] has also been shown in response to leg exercise. Our hypothesis was that both specific strength training and general fitness training would improve muscle function compared with the reference intervention. However, in the present study leg cycling did not improve muscle fiber capillarization or muscle endurance of the trapezius muscle. Thus, of the three present interventions specific strength training remains the most effective for improving muscle function in women with trapezius myalgia.

In conclusion, the case-control study showed that women with trapezius myalgia have lower strength-endurance capacity during repetitive maximal contractions of the trapezius muscle compared with healthy controls, along with lower muscle activity (EMG amplitude). Moreover, relaxation of force—measured as the rate of force relaxation—occurred more slowly in women with trapezius myalgia, although the ability to fully relax the muscles between contractions was not lower than that in healthy controls. Moreover, the intervention study demonstrated that high-intensity strength training effectively improves strength capacity during repetitive contractions of the painful trapezius muscle attaining functional levels comparable to the healthy control group together with their decrease in pain. This finding was accompanied with muscle fiber hypertrophy and increased capillarization per muscle fiber, which may at least partly explain the functional improvements. By contrast, leg cycling did not improve trapezius muscle function. Collectively, the present findings emphasize the importance of implementing specific resistance exercises in rehabilitation programmes for adults with trapezius myalgia.

## Figures and Tables

**Table 1:** Muscle fiber cross-sectional area, fiber type percentage, capillaries per fiber (CAF), and capillaries per fiber area (CAFA) in healthy controls (CON) and women with trapezius myalgia (MYA) at baseline and before and after 10-week intervention in the specific strength training (SST), general fitness training (GFT), and reference (REF) groups.

	Area (*μ*m^2^)		Fiber type percentage (%)		Capillaries per fiber (CAF)		Capillaries per fiber area (CAFA)	
	Type I	Type II	Type I	Type II	Type I	Type II	Type I	Type II
CON	5057 ± 1120	4000 ± 1104	67 ± 11	33 ± 11	4.23 ± 0.74	3.15 ± 0.72	0.89 ± 0.15	0.84 ± 0.17
MYA	5193 ± 1110	3501 ± 977	69 ± 11	31 ± 11	4.12 ± 0.87	2.79 ± 0.70	0.83 ± 0.14	0.86 ± 0.20
REF								
Before	4941 ± 399	3334 ± 489	66 ± 13	34 ± 13	3.72 ± 0.49	2.41 ± 0.33	0.81 ± 0.14	0.78 ± 0.14
After	5299 ± 1134	3471 ± 684	66 ± 14	34 ± 14	3.97 ± 0.53	2.60 ± 0.61	0.83 ± 0.18	0.78 ± 0.16
GFT								
Before	5555 ± 1201	3794 ± 796	69 ± 11	31 ± 11	4.39 ± 0.80	3.10 ± 0.51	0.85 ± 0.19	0.89 ± 0.25
After	5108 ± 1030	3612 ± 1070	72 ± 11	28 ± 11	4.18 ± 0.43	2.93 ± 0.57	0.85 ± 0.15	0.87 ± 0.34
SST								
Before	5043 ± 1294	3439 ± 1331	69 ± 12	31 ± 12	4.15 ± 1.09	2.80 ± 0.95	0.85 ± 0.13	0.88 ± 0.22
After	5516 ± 1188^#^	4133 ± 1145^$^	70 ± 10	31 ± 10	4.53 ± 0.82^#^	3.31 ± 0.64^$^	0.88 ± 0.13	0.85 ± 0.17

^#^Tendency for increase from baseline to follow-up in SST, *P* < 0.10.

^$^Significant increase from baseline to follow-up in SST, *P* < 0.05.
